# Transcriptome-wide map of m^6^A circRNAs identified in a rat model of hypoxia mediated pulmonary hypertension

**DOI:** 10.1186/s12864-020-6462-y

**Published:** 2020-01-13

**Authors:** Hua Su, Guowen Wang, Lingfang Wu, Xiuqing Ma, Kejing Ying, Ruifeng Zhang

**Affiliations:** Department of Respiratory Medicine, Sir Run Run Shaw Hospital, Zhejiang University School of Medicine, 3 East Qingchun Road, Hangzhou, China

**Keywords:** m^6^A circRNAs, Hypoxia mediated pulmonary hypertension, m^6^A circXpo6, m^6^A circTmtc3

## Abstract

**Background:**

Hypoxia mediated pulmonary hypertension (HPH) is a lethal disease and lacks effective therapy. CircRNAs play significant roles in physiological process. Recently, circRNAs are found to be m^6^A-modified. The abundance of circRNAs was influenced by m^6^A. Furthermore, the significance of m^6^A circRNAs has not been elucidated in HPH yet. Here we aim to investigate the transcriptome-wide map of m^6^A circRNAs in HPH.

**Results:**

Differentially expressed m^6^A abundance was detected in lungs of HPH rats. M^6^A abundance in circRNAs was significantly reduced in hypoxia in vitro. M^6^A circRNAs were mainly from protein-coding genes spanned single exons in control and HPH groups. Moreover, m^6^A influenced the circRNA–miRNA–mRNA co-expression network in hypoxia. M^6^A circXpo6 and m^6^A circTmtc3 were firstly identified to be downregulated in HPH.

**Conclusion:**

Our study firstly identified the transcriptome-wide map of m^6^A circRNAs in HPH.

M^6^A can influence circRNA–miRNA–mRNA network. Furthermore, we firstly identified two HPH-associated m^6^A circRNAs: circXpo6 and circTmtc3. However, the clinical significance of m^6^A circRNAs for HPH should be further validated.

## Background

Pulmonary hypertension (PH) is a lethal disease and defined as an increase in the mean pulmonary arterial pressure ≥ 25 mmHg at rest, as measured by right heart catheterization [1]. Hypoxia mediated pulmonary hypertension (HPH) belongs to group III PH according to the comprehensive clinical classification of PH, normally accompanied by severe chronic obstructive pulmonary disease (COPD) and interstitial lung diseases [2]. HPH is a progressive disease induced by chronic hypoxia [1]. Chronic hypoxia triggers over-proliferation of pulmonary artery endothelial cells (PAECs) and pulmonary artery smooth muscle cells (PASMCs), and activation of quiescent fibroblasts, the hallmark of HPH [1, 3]. The pathological characteristics of HPH are pulmonary vascular remolding, pulmonary hypertension, and right ventricular hypertrophy (RVH) [4]. So far there is no effective therapy for HPH [2]. More effective therapeutic targets are needed to be discovered.

Circular RNAs (circRNAs) were firstly found abundant in eukaryotes using RNA-seq approach [5–7]. Pre-mRNA is spliced with the 5′ and 3′ ends, forming a ‘head-to-tail’ splice junction, then circRNAs are occurred [5]. According to the genome origin, circRNAs may be classified into four different subtypes: exonic circRNA, intronic circRNA, exon–intron circRNA and tRNA introns circRNA [5]. CircRNAs are reported to play crucial roles in miRNA binding, protein binding, regulation of transcription, and post-transcription [5, 8]. Recent reports indicated that circRNAs can translate to proteins [8, 9]. Moreover, circRNAs are widely expressed in human umbilical venous endothelial cells when stimulated by hypoxia [10, 11]. Up to date, only a few reports mentioned PH-associated circRNAs. CircRNAs expression profile is demonstrated in HPH and chronic thromboembolic pulmonary hypertension [12]. However, the post-transcript modification of circRNAs in HPH is still unknown.

N^6^-methyladenosine (m^6^A) is regarded as one part of “epitranscriptomics” and identified as the most universal modification on mRNAs and noncoding RNAs (ncRNAs) in eukaryotes [13, 14]. DRm^6^ACH (D denotes A, U or G; R denotes A, G; H denotes A, C, or U) is a consensus motif occurred in m^6^A modified RNAs [15–17]. M^6^A modification is mainly enriched around the stop codons, at 3’untranslated regions and within internal long exons [17–19]. Several catalyzed molecules act as “writers”, “readers”, and “erasers” to regulate the m^6^A modification status [14]. The methyltransferase complex is known as writers, including methyltransferase-like-3, − 14 and − 16 (METTL3/METTL14/METTL16), Wilms tumor 1-associated protein (WTAP), RNA binding motif protein 15 (RBM15), vir like m^6^A methyltransferase associated (KIAA1429) and zinc finger CCCH-type containing 13 (ZC3H13), appending m^6^A on DRACH [17, 20, 21]. METTL3 is regarded as the core catalytically active subunit, while METTL14 and WTAP play a structural role in METTL3’s catalytic activity [18, 22]. The “erasers”, fat mass and obesity related protein (FTO) and alkylation repair homolog 5 (ALKBH5), catalyze the N-alkylated nucleic acid bases oxidatively demethylated [22]. The “readers”, the YT521-B homology (YTH) domain-containing proteins family includes YTHDF (YTHDF1, YTHDF2, YTHDF3), YTHDC1, and YTHDC2, specifically recognizes m^6^A and regulates splicing, localization, degradation and translation of RNAs [14, 22, 23]. The YTHDF1 and YTHDF2 crystal structures forms an aromatic cage to recognize m^6^A sites in cytoplasm [24]. YTHDC1 is the nuclear reader and YTHDC2 binds m^6^A under specific circumstances or cell types [24]. Hypoxia may alter the balance of writers-erasers-readers and induce tumor growth, angiogenesis, and progression [25, 26].

Interestingly, circRNAs can be m^6^A-modified. M^6^A circRNAs displayed cell-type-specific methylation patterns in human embryonic stem cells and HeLa cells [14]. CircRNAs contained m^6^A modifications are likely to promote protein translation in a cap-independent pattern [9]. However, m^6^A circRNAs has not been elucidated in HPH yet. Here we are the first to identify the expression profiling of m^6^A circRNAs in HPH.

## Results

### M^6^A level of circRNAs was reduced in HPH rats and most circRNAs contained one m^6^A peak

**Fig. 1:**
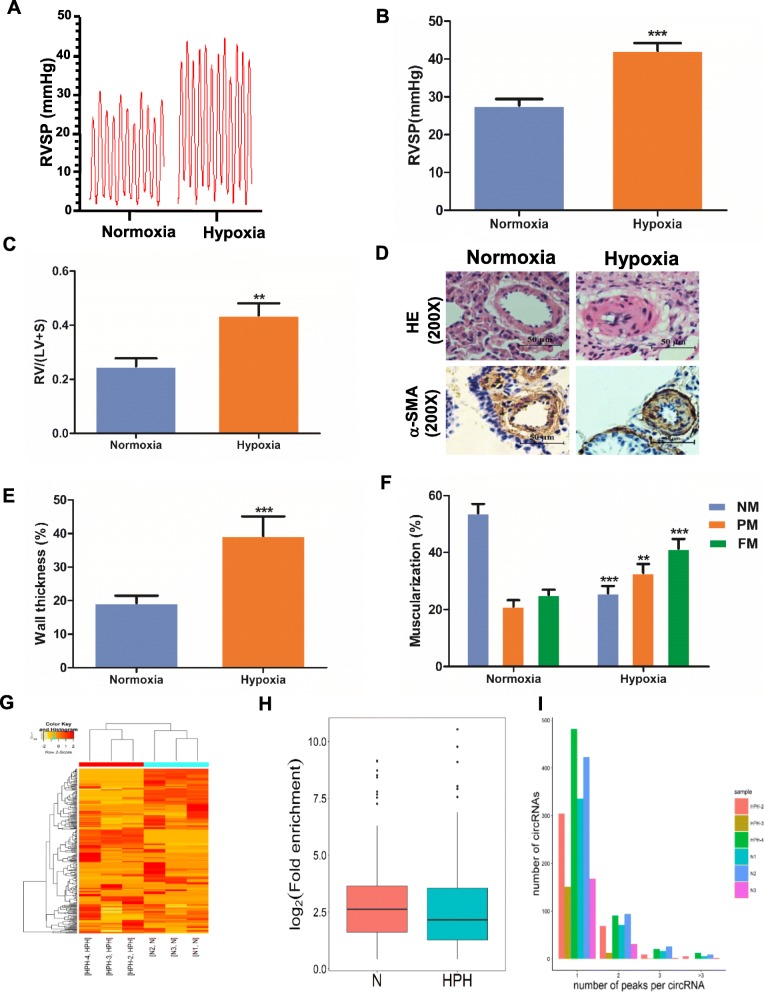
M^6^A level of circRNAs in HPH rats and the number of m^6^A peak in circRNAs Rats were maintained in a normobaric normoxic (N, FiO_2_ 21%) or hypoxic (HPH, FiO_2_ 10%) chamber for 3 weeks, then RVSP was detected (**a, b**). **c** The ratio of RV/ (LV + S). **d** H&E staining and immunohistochemical staining of α-SMA were performed in the lung sections. Representative images of pulmonary small arteries. Scale bar = 50 μm. Quantification of wall thickness (**e**) and vessel muscularization (**f**). **g** Heatmap depicting hierarchical clustering of altered m^6^A circRNAs in lungs of N and HPH rats. Red represents higher expression and yellow represents lower expression level. **h** Box-plot for m^6^A peaks enrichment in circRNAs in N and HPH. **i** Distribution of the number of circRNAs (y axis) was plotted based on the number of m^6^A peaks in circRNAs (x axis) in N and HPH. Values are presented as means ± SD (*n* = 6 in each group). Only vessels with diameter between 30 and 90 μm were analyzed. NM, nonmuscularized vessels; PM, partially muscularized vessels; FM, fully muscularized vessels. **0.001 ≤ *P* ≤ 0.009 (different from N); ***P < 0.001 (different from N)

Three weeks treatment by hypoxia resulted in right ventricular systolic pressure (RVSP) elevating to 42.23 ± 1.96 mmHg compared with 27.73 ± 1.71 mmHg in the control (*P* < 0.001, Fig. 1a and b). The ratio of the right ventricle (RV), left ventricular plus ventricular septum (LV + S) [RV/ (LV + S)] was used as an index of RVH. RVH was indicated by the increase of RV/ (LV + S) compared with the control (0.25 ± 0.03 vs. 0.44 ± 0.04, *P* = 0.001, Fig. 1c). The medial wall of the pulmonary small arteries was also significantly thickened (19.28 ± 2.19% vs. 39.26 ± 5.83%, *P* < 0.001, Fig. 1d and e). Moreover, in the normoxia group, 53.82 ± 3.27% of the arterioles were non-muscularized (NM) vessels, and 25.13 ± 1.83% were fully muscularized (FM) vessels. In contrast, partially muscularized vessels (PM) and FM vessels showed a greater proportion (32.88 ± 3.15% and 41.41 ± 3.35%) in HPH rats, while NM vessels occupied a lower proportion (25.71 ± 2.55%) (Fig. 1f). Figure 1g displayed the heatmap of m^6^A circRNAs expression profiling in N and HPH. M^6^A abundance in 166 circRNAs was significantly upregulated. Meanwhile, m^6^A abundance in 191 circRNAs was significantly downregulated (Additional file 1: Data S1, filtered by fold change ≥4 and *P* ≤ 0.00001). Lungs of N and HPH rats were selected to measure m^6^A abundance in purified circRNAs. The m^6^A level in total circRNAs isolated from lungs of HPH rats was lower than that from controls (Fig. 1h). Moreover, over 50% circRNAs contained only one m^6^A peak either in lungs of N or HPH rats (Fig. 1i).

### M^6^A circRNAs were mainly from protein-coding genes spanned single exons in N and HPH groups

**Fig. 2:**
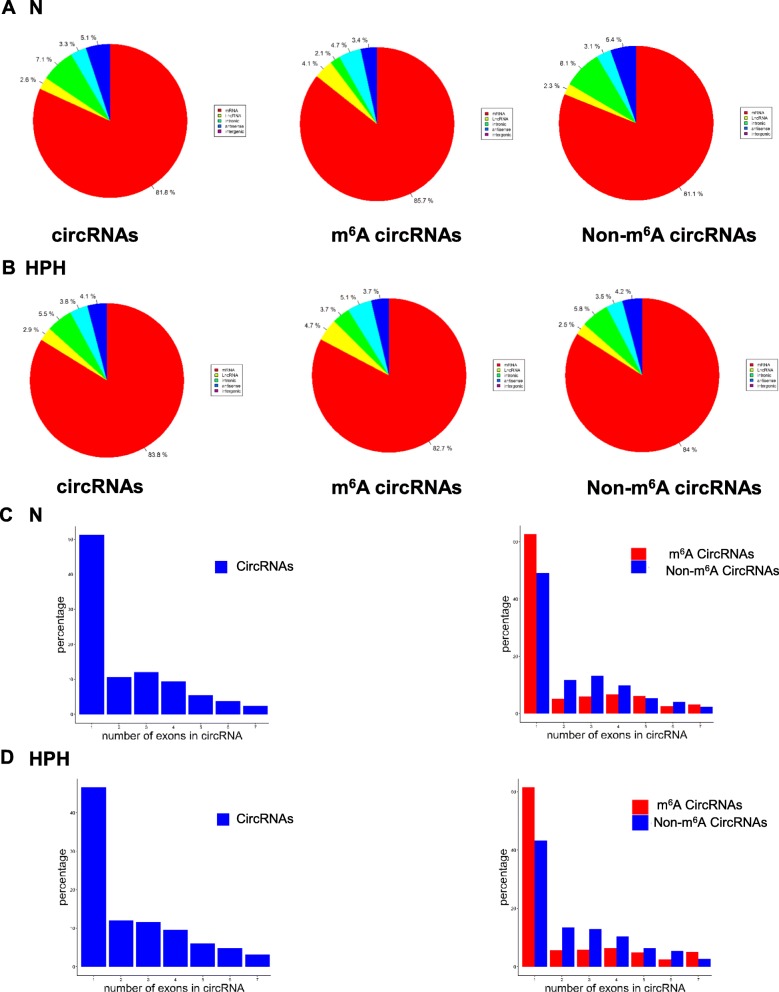
The genomic origins of m^6^A circRNAs The distribution of genomic origins of total circRNAs (input, left), m^6^A circRNAs (eluate, center), and non-m^6^A circRNAs (supernatant, right) in N (**a**) and HPH (**b**). The percentage of circRNAs (y axis) was calculated according to the number of exons (x axis) spanned by each circRNA for the input circRNAs (left), m^6^A-circRNAs (red, right) and non-m^6^A circRNAs (blue, right) in N (**c**) and HPH (**d**). Up to seven exons are shown

We analyzed the distribution of the parent genes of total circRNAs, m^6^A-circRNAs, and non-m^6^A circRNAs in N and HPH, respectively. N and HPH groups showed a similar genomic distribution of m^6^A circRNAs and non-m^6^A circRNAs (Fig. 2a and b). Moreover, about 80% of m^6^A circRNAs and non-m^6^A circRNAs were derived from protein-coding genes in both groups. A previous report indicated that most circRNAs originated from protein-coding genes spanned two or three exons [14]. While in our study, over 50 and 40% of total circRNAs from protein-coding genes spanned one exon in N and HPH groups, respectively (Fig. 2c and d**)**. Similarly, m^6^A circRNAs and non-m^6^A circRNAs were mostly encoded by single exons. Therefore, it was indicated that m^6^A methylation was abundant in circRNAs originated from single exons in N and HPH groups.

### The distribution and functional analysis for host genes of circRNAs with differentially expressed m^6^A peaks

**Fig. 3:**
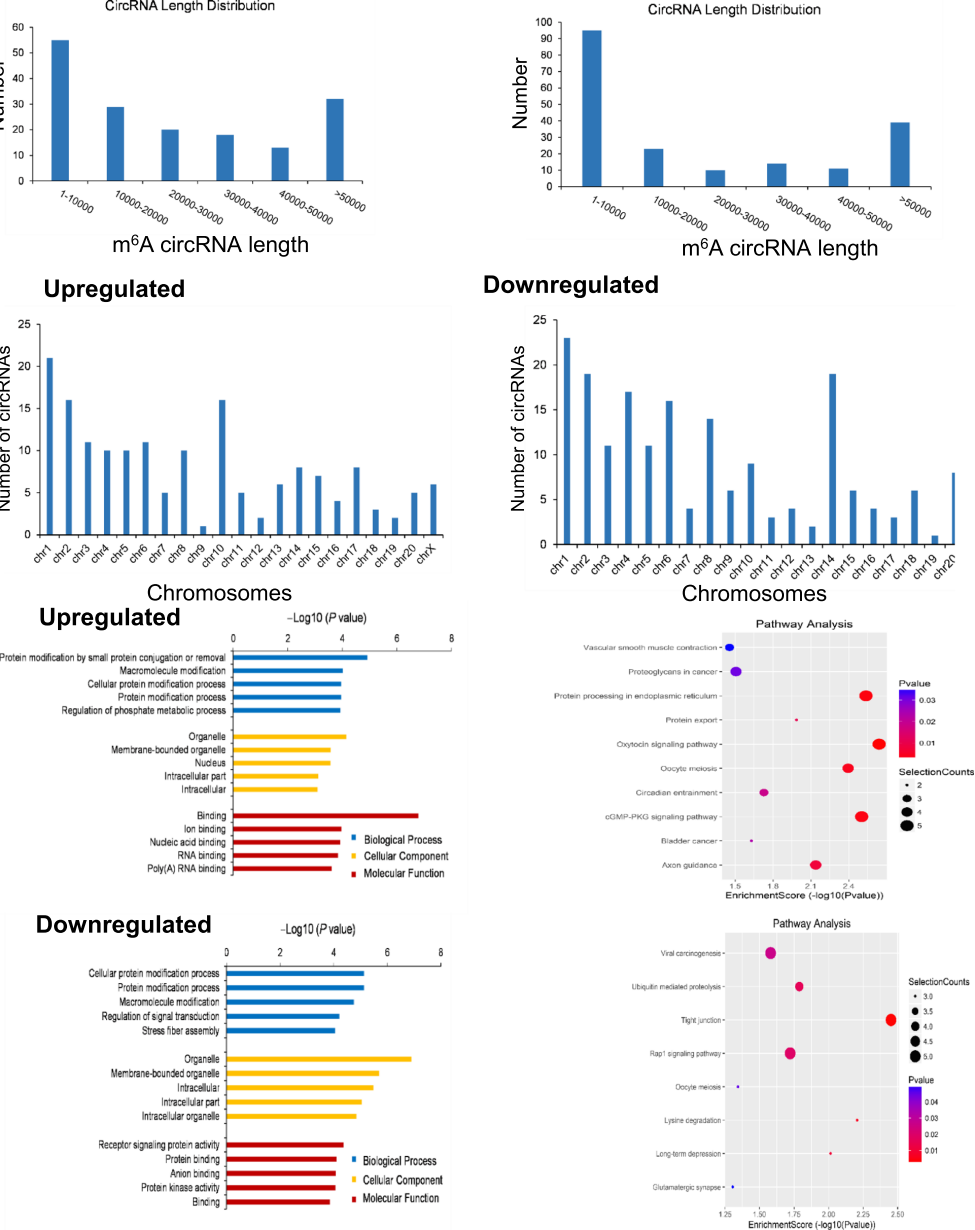
The distribution and functional analysis for host genes of circRNAs with differentially expressed (DE) m^6^A peaks (**a**) Length of DE m^6^A circRNAs. **b** The chromosomes origins for host genes of DE m^6^A circRNAs. GO enrichment and KEGG signaling pathway analysis for host genes of upregulated (**c**) and downregulated (**d**) m^6^A circRNAs. GO enrichment analysis include biological process (BP) analysis, cellular component (CC) analysis, and molecular function (MF) analysis. *P* values are calculated by DAVID tool

The length of differentially-expressed m^6^A circRNAs was mostly enriched in 1–10,000 bps (Fig. 3a). The host genes of upregulated m^6^A circRNAs were located in chromosome 1, 2 and 10, while the downregulated parts were mostly located in chromosome 1, 2 and 14 (Fig. 3b).

Gene ontology (GO) analysis and Kyoto Encyclopedia of Genes and Genomes (KEGG) pathway analysis were performed to explore the host genes of circRNAs with differentially-expressed m^6^A peaks. In the GO analysis (Fig. 3c, left), the parent genes of circRNAs with upregulated m^6^A peaks were enriched in the protein modification by small protein conjugation or removal and macromolecule modification process in the biological process (BP). Organelle and membrane-bounded organelle were also the two largest parts in the cellular component (CC) analysis. Binding and ion binding were the two main molecular functions (MF) analysis. The top 10 pathways from KEGG pathway analysis were selected in the bubble chart (Fig. 3c, right). Among them, the oxytocin signaling pathway, protein processing in endoplasmic reticulum and cGMP-PKG signaling pathway were the top 3 pathways involved. In addition, vascular smooth muscle contraction pathway was the most associated pathway in PH progression [27].

In Fig. 3d left, the parent genes of circRNAs with downregulated m^6^A peaks were mainly enriched in the cellular protein modification process and protein modification process in BP. Organelle and membrane-bounded organelle made up the largest proportion in the CC classification. The MF analysis was focused on receptor signaling protein activity and protein binding. The parent genes of circRNAs with decreased m^6^A peaks were mainly involved in the tight junction and lysine degradation in the KEGG pathway analysis (Fig. 3d, right).

### Hypoxia can influence the m^6^A level of circRNAs and circRNAs abundance

**Fig. 4:**
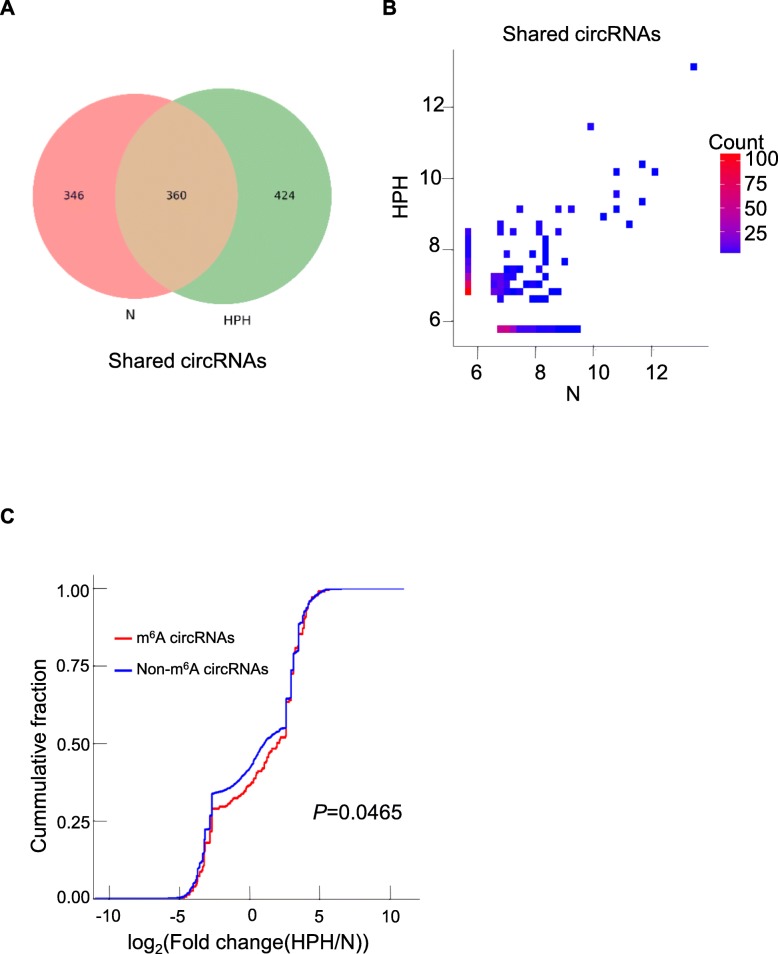
The relationship of m^6^A level and circRNAs abundance in hypoxia (**a**) Venn diagram depicting the overlap of m^6^A circRNAs between N and HPH. **b** Two-dimensional histograms comparing the expression of m^6^A circRNAs in lungs of N and HPH rats. It showed that m^6^A circRNAs levels for all shared circRNAs in both groups. CircRNAs counts were indicated on the scale to the right. **c** Cumulative distribution of circRNAs expression between N and HPH for m^6^A circRNAs (red) and non-m^6^A circRNAs (blue). *P* value was calculated using two-sided Wilcoxon-Mann-Whiteney test

360 m^6^A circRNAs were shared in N and HPH groups. 49% of m^6^A circRNAs detected in N group were not detected in HPH group, and 54% of m^6^A circRNAs detected in HPH group were not detected in N group (Fig. 4a). To explore whether m^6^A methylation would influence circRNAs expression level, expression of the 360 common m^6^A circRNAs were identified. More circRNAs tended to decrease in HPH compared to N (Fig. 4b). Moreover, expression of m^6^A circRNAs was significantly downregulated compared with non-m^6^A circRNAs in hypoxia, suggesting that m^6^A may downregulate the expression of circRNAs in hypoxia (Fig. 4c**,** *P* = 0.0465).

### Construction of a circRNA–miRNA–mRNA co-expression network in HPH

**Fig. 5:**
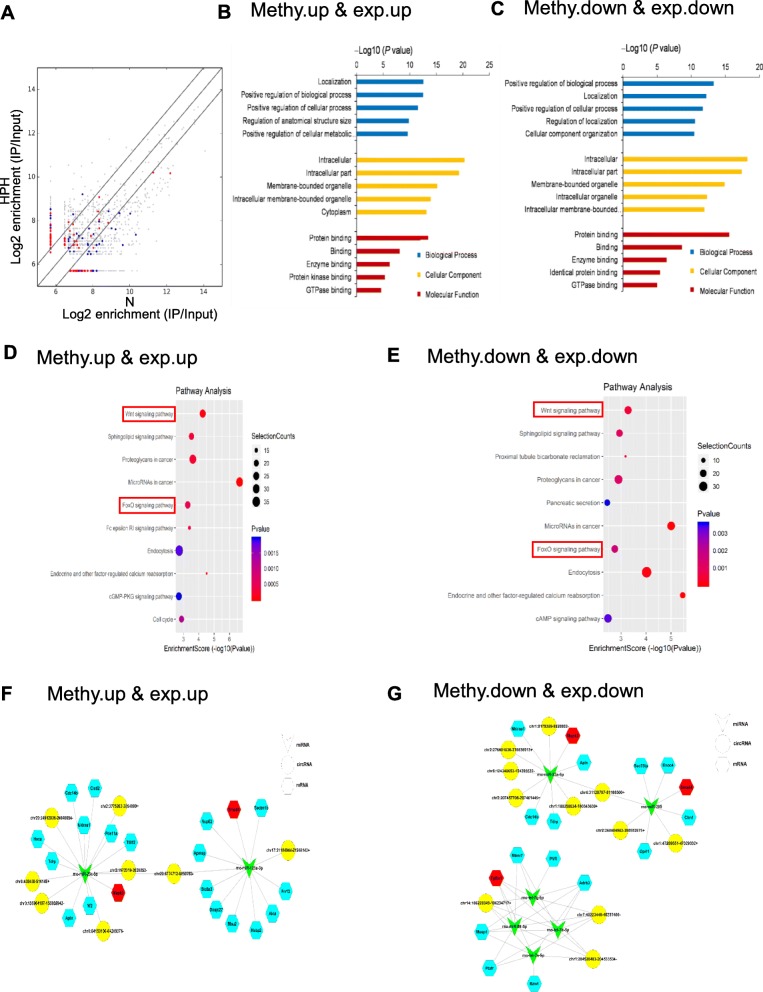
Construction of a circRNA–miRNA–mRNA co-expression network in HPH (**a**) Comparison of the relationship between m^6^A level and expression of circRNAs between N and HPH. The fold-change ≥2.0 was considered to be significant, which was the m^6^A abundance of HPH relative to N. Red dots represents circRNAs with upregulated m^6^A level and blue dots represents circRNAs with downregulated m^6^A level. IP/Input referred to the m^6^A abundance in circRNAs detected in MeRIP-Seq (IP) normalized to that detected in input. **b** and **c** GO enrichment analysis includes BP analysis, CC analysis, and MF analysis. *P* values are calculated by DAVID tool. **d** and **e** KEGG signaling pathway analysis for the downstream mRNAs which was predicted to be ceRNA of DE cirRNAs. Methy. down & exp. down represents downregulated cirRNAs with decreased m^6^A level. Methy. up & exp. up represents upregulated cirRNAs with increased m^6^A level. **f** and **g** CeRNA analysis for DE circRNAs. Network map of circRNA-miRNA-mRNA interactions. Green V type node: miRNA; yellow circular node: DE circRNAs; blue hexagon node: target genes of miRNAs; red hexagon node: PH-related genes

We found 76 upregulated circRNAs with increased m^6^A abundance, and 107 downregulated circRNAs with decreased m^6^A abundance (Fig. 5a**,** Additional file 2: Data S2, Additional files 3 and 4). As known, circRNAs were mostly regarded as a sponge for miRNAs and regulated the expression of corresponding target genes of miRNAs [28]. To explore whether circRNAs with differentially-expressed m^6^A abundance influence the availability of miRNAs to target genes, we selected differentially-expressed circRNAs with increased or decreased m^6^A abundance. GO enrichment analysis and KEGG pathway analysis were also performed to analyze target mRNAs. Target mRNAs displayed similar GO enrichment in the two groups (Fig. 5b and c). Two main functions were determined in BP analysis: positive regulation of biological process and localization. Intracellular and intracellular parts make up the largest proportion in CC part. Target mRNAs were mostly involved in protein binding and binding in MF part. In the KEGG pathway analysis, the top 10 most enriched pathways were selected (Fig. 5d and e). Wnt and FoxO signaling pathways were reported to be involved in PH progression [29–31]. Then, we analyzed the target genes involved in these two pathways. SMAD4 was associated with PH and involved in Wnt signaling pathways. MAPK3, SMAD4, TGFBR1, and CDKN1B were involved in FoxO signaling pathways. To explore the influence of circRNA-miRNA regulation on PH-associated genes expression, we constructed a circRNA-miRNA-mRNA network, integrating matched expression profiles of circRNAs, miRNAs and mRNAs (Fig. 5f and g). MicroRNAs sponged by the target genes of interest were analyzed. MiR-125a-3p, miR-23a-5p, miR-98-5p, let-7b-5p, let-7a-5p, let-7 g-5p, and miR-205 were analyzed because they were reported to be associated with PH [32, 33]. We filtered the key mRNAs and miRNAs, and founded that the two circRNAs were the most enriched, which were originated from chr1:204520403–204,533,534- (Xpo6) and chr7:40223440–40,237,400- (Tmtc3).

### M^6^A circXpo6 and m^6^A circTmtc3 were downregulated in PASMCs and PAECs in hypoxia

**Fig. 6:**
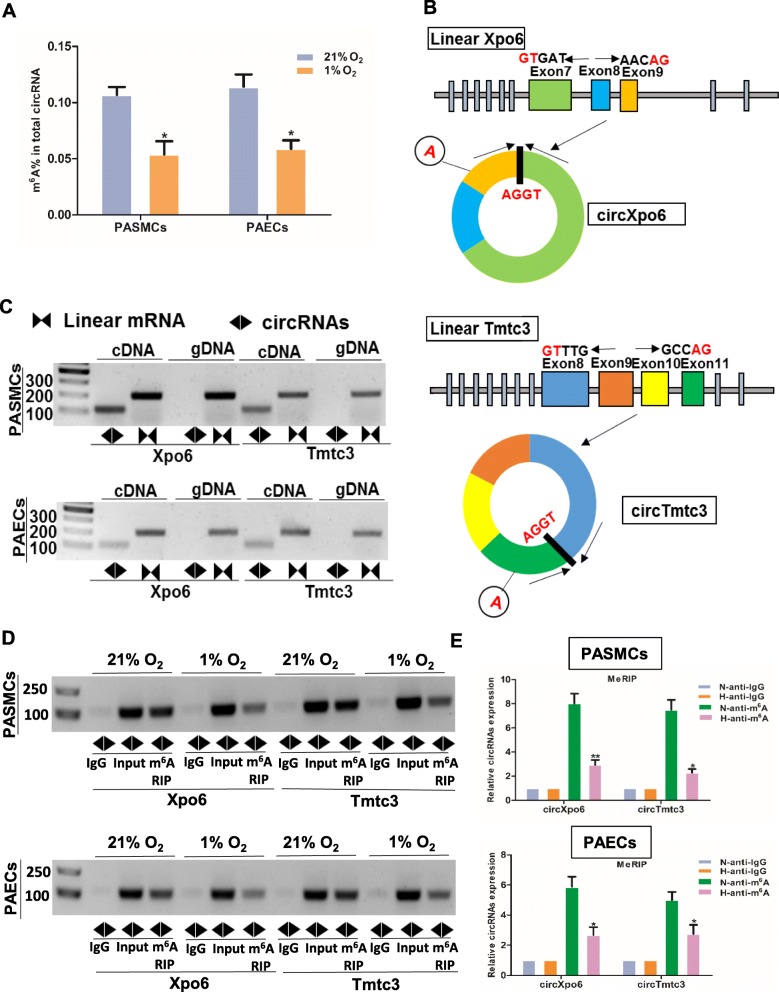
The expression profiling of m^6^A circXpo6 and m^6^A circTmtc3 in pulmonary arterial smooth muscle cells (PASMCs) and pulmonary artery endothelial cells (PAECs) in hypoxia. **a** M^6^A levels of total circRNAs were determined based on colorimetric method in vitro. PASMCs and PAECs were exposed to 21% O_2_ and 1% O_2_ for 48 h, respectively. Total RNA was extracted and treated by RNase R. M^6^A levels were determined as a percentage of total circRNAs. **b** Schematic representation of exons of the Xpo6 and Tmtc3 circularization forming circXpo6 and circTmtc3 (black arrow). **c** RT-PCR validation of circXpo6 and circTmtc3 in PASMCs and PAECs exposed to 21% O_2._ Divergent primers amplified circRNAs in cDNA, but not in genomic DNA (gDNA). The size of the DNA marker is indicated on the left of the gel. **d** and **e** RT-PCR and qRT-PCR was performed after m^6^A RIP in PASMCs and PAECs exposed to 21% (N) and 1% O_2_ (H) for 48 h, respectively. Input was used as a control (**d**). IgG was used as a negative control (**d** and **e**). Values are presented as means ± SD. *P ≤ 0.05 (different from 21% O_2_ or the N-anti-m^6^A); **0.001 ≤ *P* ≤ 0.009 (different from the N-anti-m^6^A), (*n* = 3 each)

M^6^A abundance was significantly reduced in PASMCs and PAECs when exposed to hypoxia (0.107% ± 0.007 vs. 0.054% ± 0.118, *P* = 0.023 in PASMCs; 0.114% ± 0.011 vs. 0.059% ± 0.008, *P* = 0.031 in PAECs, Fig. 6**a**). M^6^A abundance in circRNAs was lower than it in mRNAs (0.1–0.4%) [17, 18]. Next, we confirmed the back-splicing of circXpo6 and circTmtc3 by CIRI software. The sequence of linear Xpo6 and Tmtc3 mRNA was analyzed. Then we identified that circXpo6 was spliced form exon 7, 8, and 9 of Xpo6. CircTmtc3 was spliced form exon 8, 9, 10, and 11 (Fig. 6b). Using cDNA and genomic DNA (gDNA) from PASMCs and PAECs as templates, circXpo6 and circTmtc3 were only amplified by divergent primers in cDNA, while no product was detected in gDNA (Fig. 6c). To identify whether circXpo6 and circTmtc3 were modified by m^6^A, we performed M^6^A RNA Immunoprecipitation (MeRIP)-RT-PCR and MeRIP-quantitative RT-PCR (MeRIP-qRT-PCR) to detect the expression of circXpo6 and circTmtc3 (Fig. 6d and e). m^6^A circXpo6 and m^6^A circTmtc3 were significantly decreased in PASMCs and PAECs when exposed to hypoxia (*P* = 0.002, and *P* = 0.015 in PASMCs and *P* = 0.02, and *P* = 0.047 in PAECs).

## Discussion

In this study, we identified the transcriptome-wide map of m^6^A circRNAs in hypoxia mediated pulmonary hypertension. On the whole, we found that m^6^A level in circRNAs was reduced in lungs when exposed to hypoxia. M^6^A circRNAs were mainly derived from single exons of protein-coding genes in N and HPH. M^6^A abundance in circRNAs was downregulated in hypoxia in vitro. M^6^A influenced the circRNA–miRNA–mRNA co-expression network in hypoxia. Moreover, circXpo6 and circTmtc3 were the novel identified circRNAs modified by m^6^A in hypoxia mediated pulmonary hypertension.

M^6^A plays important roles in various biological processes. M^6^A is associated with cancer progression, promoting the proliferation of cancer cells and contributing to the cancer stem cell self-renewal [18, 21]. Lipid accumulation was reduced in hepatic cells when m^6^A abundance in peroxisome proliferator-activator (*PPaR)* was decreased [34]. Enhanced m^6^A level of mRNA contributed to compensated cardiac hypertrophy [35]. Also, m^6^A modification of large intergenic noncoding RNA 1281 was necessary for mouse embryonic stem cells differentiation [36].

Although it has been reported that m^6^A mRNAs were influenced by hypoxia, there is no report about m^6^A circRNAs in HPH yet. Up to now, no consistent conclusion was reached about the link between m^6^A and hypoxia. Previous reports found that the m^6^A abundance in mRNA was increased under hypoxia stress in HEK293T cells and cardiomyocytes [37, 38]. The increased m^6^A level stabilized the mRNAs of Glucose Transporter 1 (Glut1), Myc proto-oncogene bHLH transcription factor (Myc), Dual Specificity Protein Phosphatase 1 (Dusp1), Hairy and Enhancer of Split 1 (Hes1), and Jun Proto-Oncogene AP-1 Transcription Factor Subunit (Jun) without influencing their protein level [37]. In contrast, another reported that m^6^A level of total mRNA was decreased when human breast cancer cell lines were exposed to 1% O_2_ [26]. Hypoxia increased demethylation by stimulating hypoxia-inducible factor (HIF)-1α- and HIF-2α–dependent over-expression of ALKBH5 [26]. In addition, transcription factor EB activates the transcription of ALKBH5 and downregulates the stability of METTL3 mRNA in hypoxia/reoxygenation-induced autophagy in ischemic diseases [38]. Our study found that m^6^A abundance in total circRNAs was decreased in hypoxia exposure. Moreover, our study indicated that circXpo6 and circTmtc3 were the novel identified circRNAs modified by m^6^A in HPH. M^6^A abundance in circXpo6 and circTmtc3 was decreased in hypoxia. It is probably because of HIF-dependent and ALKBH5-mediated m^6^A demethylation [26].

Previous reports indicated that m^6^A methylation close to 3’UTR and stop codon of mRNA is inversely correlated with gene expression [14, 39]. Low m^6^A level is negatively associated with circRNAs expression, while high m^6^A level is not linked to circRNAs expression in human embryonic stem cells and HeLa cells [14]. Consistent with the previous reports [14, 39], our study found that m^6^A reduced the total circRNAs abundance in hypoxia. The association between m^6^A level and specific gene abundance is remained as an open question. Some previous reports indicated that m^6^A level was positively associated with long non-coding RNA (lncRNA) or mRNA expression [40, 41]. M^6^A was positively associated with RP11–138 J23.1 (RP11) expression when ALKBH5 was overexpressed in colorectal cancer [40]. mRNAs were downregulated after METTL14 deletion in β-cells [41]. On the contrary, another reports insisted that m^6^A level was negatively associated with RNA expression [42–44]. the mRNA lifetime of Family with Sequence Similarity 134, Member B (FAM134B) was prolonged when the m^6^A site was mutant [42]. The decreased m^6^A level resulted in the increased expression of N-methyl-D-aspartate receptor 1 (NMDAR1) in Parkinson’s disease [43]. Forkhead Box protein M1 (FOXM1) abundance was increased when ALKBH5 was upregulated in glioblastoma [44]. Our study indicated that the expression of circXpo6 and circTmtc3 was decreased with the downregulated m^6^A level. The association between m^6^A level and circRNAs abundance was not determined yet. We suspected that m^6^A may influence the expression of circXpo6 and circTmtc3 through similar manners as before [40, 41]. But it needs further validation.

Competing endogenous RNA (CeRNA) mechanism was proposed that mRNAs, pseudogenes, lncRNAs and circRNAs interact with each other by competitive binding to miRNA response elements (MREs) [45, 46]. M^6^A acts as a post-transcript regulation of circRNAs and influences circRNAs expression, thus we suggested that m^6^A could also regulate the circRNA–miRNA–mRNA co-expression network. When the circRNAs were classified, we found that these downstream targets regulated by circRNA–miRNA of interest were mostly enriched in PH-associated Wnt and FoxO signaling pathways [30, 31]. The Wnt/β-catenin (bC) pathway and Wnt/ planar cell polarity (PCP) pathway are the two most critical Wnt signaling pathways in PH [30]. As known, the two important cells associated with HPH are PASMCs and PAECs [1, 3]. The growth of PASMCs was increased when Wnt/bC and Wnt/PCP pathways were activated by platelet derived growth factor beta polypeptide b (PDGF-BB) [30, 47]. In addition, the proliferation of PAECs was enhanced when Wnt/bC and Wnt/PCP pathways were activated by bone morphogenetic protein 2 (BMP2). Furthermore, the FoxO signaling pathway is associated with the apoptosis-resistant and hyper-proliferative phenotype of PASMCs [31]. Reactive oxygen species is increased by hypoxia and activates AMPK-dependent regulation of FoxO1 expression, resulting in increased expression of catalase in PASMCs [48]. Our study firstly uncovered that m^6^A influenced the stability of circRNAs, thus affecting the binding of circRNAs and miRNA, resulting in the activation of Wnt and FoxO signaling pathways.

## Conclusion

In conclusion, our study firstly identified the transcriptome-wide map of m^6^A circRNAs in HPH. M^6^A level in circRNAs was decreased in lungs of HPH and in PASMCs and PAECs exposed to hypoxia. M^6^A level influenced circRNA–miRNA–mRNA co-expression network in HPH. Moreover, we firstly identified two downregulated m^6^A circRNAs in HPH: circXpo6 and circTmtc3. CircRNAs may be used as biomarkers because it is differentially enriched in specific cell types or tissues and not easily degraded [6]. Also, the aberrant m^6^A methylation may contribute to tumor formation and m^6^A RNAs may be a potential therapy target for tumor [17].

Limitations still exist in the study. First, we did not analyze the m^6^A level between circRNAs and the host genes. Second, the exact mechanism of hypoxia influences m^6^A was not demonstrated. Thirdly, the function of circXpo6 and circTmtc3 in HPH was not elaborated. Lastly, besides hypoxia mediated pulmonary hypertension, many other significant PH models should also be noted, such as monocrotaline mediated PH, monocrotaline + pneumonectomy mediated PH, and so on. It is insufficient that we explored the expression profiling of m^6^A circRNAs only in hypoxia mediated pulmonary hypertension. We plan to explore the expression profiling of m^6^A circRNAs in monocrotaline-induced PH and other PH models. Moreover, the clinical significance of m^6^A circRNAs for HPH should be further validated.

## Methods

### Hypoxia mediated PH rat model and measurement of RVSP and RVH

Sprague-Dawley rats (SPF, male, 180–200 g, 4 weeks) were obtained from the Animal Experimental Center of Zhejiang University, China. Rats were maintained in a normobaric normoxia (FiO_2_ 21%, *n* = 6) or hypoxic chamber (FiO_2_ 10%, *n* = 6) for 3 weeks [3, 49]. Rats were then anesthetized by intraperitoneal injection of 1% sodium pentobarbital (130 mg/kg) [50]. Then, rats were fixed in supine position on the board. All of the operations were performed after rats were anesthetized and became unconscious. RVSP was measured as below. Right ventricle catheterization was performed through the right jugular using a pressure-volume loop catheter (Millar) as the previous reports [49, 51]. After measurement of RVSP, all rats were put into a confined and transparent euthanasia device (to observe whether the rats were sacrificed), then 100% CO_2_ was released into the device continuously until all the rats sacrificed. The criteria for sacrifice were that rats did not have spontaneous breath for 2–3 min and blink reflex. Then, heart tissues were removed and segregated. The ratio of [RV/ (LV + S)] was used as an index of RVH. Lung were removed and immediately frozen at liquid nitrogen or fixed in 4% buffered paraformaldehyde solution. All experimental procedures were conducted in line with the principles approved by the Institutional Animal Care and Use Committee of Zhejiang University.

### Histological analysis

Lung tissues were embedded in paraffin, sectioned at 4 μm and stained with hematoxylin and eosin (H&E) and α-smooth muscle actin (α-SMA, 1:100, ab124964, Abcam, USA). The ratio of pulmonary small artery wall thickness and muscularization were calculated [3].

### Isolation and hypoxia-treatment of PASMCs and PAECs

PASMCs and PAECs were isolated using the methods according to previous reports [32, 50, 52]. PASMCs and PAECs were cultured in Dulbecco’s modified Eagle’s medium supplemented with 10% fetal bovine serum (FBS) and 20% FBS for 48 h, respectively [32, 53]. The cells were incubated in a 37 °C, 21% O_2_ or 1% O_2_–5% CO_2_ humidified incubator. PASMCs at 70–80% confluence in 4 to 7 passages were used in experiments. PAECs at 80–90% confluence in 4 to 5 passages were used in experiments [54].

### RNA isolation and RNA-seq analysis of circRNAs

Total RNA (10 mg) was obtained using TRIzol reagent (Invitrogen, Carlsbad, CA, USA) from lungs (1 g) of control and HPH rats. The extracted RNAs were purified with Rnase R (RNR07250, Epicentre) digestion to remove linear transcripts. Paired-end reads were harvested from Illumina Hiseq Sequence after quality filtering. The reads were aligned to the reference genome (UCSC RN5) with STAR software. CircRNAs were detected and annotated with CIRI software [55]. Raw junction reads were normalized to per million number of reads mapped to the genome with log2 scaled.

### MeRIP and library preparation

Total RNA was extracted as the methods described above. Then, rRNA was depleted following DNase I treatment. RNase R treatment (5 units/mg) was performed in duplicate with 5 mg of rRNA-depleted RNA input. High-throughput m^6^A and circRNAs sequencing were performed by Cloudseq Biotech Inc. (Shanghai, China). Fragmented RNA was incubated with anti-m^6^A polyclonal antibody (Synaptic Systems, 202,003) in IPP buffer for 2 h at 4 °C. The mixture was then incubated with protein A/G magnetic beads (88,802, Thermo Fisher) at 4 °C for an additional 2 h. Then, bound RNA was eluted from the beads with N^6^-methyladenosine (PR3732, BERRY & ASSOCIATES) in IPP buffer and extracted with Trizol reagent (15,596,026, Thermo Fisher). NEBNext® Ultra™ RNA Library Prep Kit (E7530L, NEB) was used to construct RNA-seq library from immunoprecipitated RNA and input RNA. The m^6^A-IP and input samples were subjected to 150 bp paired-end sequencing on Illumina HiSeq sequencer. Methylated sites on circRNAs were identified by MetPeak software.

### Construction of circRNA–miRNA–mRNA co-expression network

The circRNA–miRNA–mRNA co-expression network was based on the ceRNA theory that circRNA and mRNA shared the same MREs [45, 46]. Cytoscape was used to visualize the circRNA–miRNA–mRNA interactions based on the RNA-seq data. The circRNA-miRNA interaction and miRNA–mRNA interaction of interest were predicted by TargetScan and miRanda.

### Measurement of Total m^6^A, MeRIP-RT-PCR and MeRIP-qRT-PCR

Total m^6^A content was measured in 200 ng aliquots of total RNA extracted from PASMCs and PAECs exposed to 21% O_2_ and 1% O_2_ for 48 h using an m^6^A RNA methylation quantification kit (P-9005, Epigentek). MeRIP (17–701, Millipore) was performed according to the manufacturer’s instruction. A 1.5 g aliquot of anti-m^6^A antibody (ABE572, Millipore) or anti-IgG (PP64B, Millipore) was conjugated to protein A/G magnetic beads overnight at 4 °C. A 100 ng aliquot of total RNA was then incubated with the antibody in IP buffer supplemented with RNase inhibitor and protease inhibitor. The RNA complexes were isolated through phenol-chloroform extraction (P1025, Solarbio) and analyzed via RT-PCR or qRT-PCR assays. Primers sequences are listed in Table 1.

**Table 1:** Primers for RT-PCR or qRT-PCR

Name	Sequence	Product size(bp)
linear Xpo6	Sense: 5’CTGTGTTTTGGGTCAGGAGC3’ Antisense: 5’ATCGAGTTCCTCTAGCCTGC3’	199
linear Tmtc3	Sense: 5’ACTCTGCTGTGATTGGACCA3’ Antisense:5’AGAAGAGGTTTGATGCGGGA3’	203
circXpo6	Sense: 5’TCTGGGAGACAAGGAAGCAG3’ Antisense: 5’CAGGATGGGGATGGGCTG3’	101
circTmtc3	Sense: 5’TACCCATGTTCAGCCAGGTT3’ Antisense:5’GAAGCCAAGCATTCACAGGA3’	100

### Data analysis

3′ adaptor-trimming and low quality reads were removed by cutadapt software (v1.9.3). Differentially methylated sites were identified by the R MeTDiff package. The read alignments on genome could be visualized using the tool IGV. Differentially expressed circRNAs were identified by Student’s *t*-test. GO and KEGG pathway enrichment analysis were performed for the corresponding parental mRNAs of the DE circRNAs. GO enrichment analysis was performed using the R topGO package. KEGG pathway enrichment analysis was performed according to a previous report [56]. GO analysis included BP analysis, CC analysis, and MF analysis. MicroRNAs sponged by the target genes were predicted by TargetScan and microRNA websites. *P* values are calculated by DAVID tool for GO and KEGG pathway analysis. The rest statistical analyses were performed with SPSS 19.0 (Chicago, IL, USA) and GraphPad Prism 5 software (La Jolla, CA). N refers to number of samples in figure legends. The statistical significance was determined by Student’s *t*-test (two-tailed) or two-sided Wilcoxon-Mann-Whiteney test. *P* < 0.05 was considered statistically significant. All experiments were independently repeated at least three times.

## Supplementary information

**Additional file 1: Data S1.** Differentially expressed m^6^A abundance in circRNAs.

**Additional file 2: Data S2.** Differentially expressed m^6^A abundance linked with differentially expressed circRNAs abundance.

**Additional file 3.** Differentially expressed m^6^A circRNAs in the lungs of HPH rat model.

**Additional file 4.** Differentially expressed circRNAs in the lungs of HPH rat model.

## Data Availability

The raw high-throughput m^6^A and circRNAs sequencing generated during the current study has been uploaded into the repository named Zenodo, the persistent web links are 10.5281/zenodo.3596039, 10.5281/zenodo.3596178, 10.5281/zenodo.3596016, and 10.5281/zenodo.3596276.

## Abbreviations

ALKBH5: Alkylation repair homolog 5
BMP2: Bone morphogenetic protein 2
BP: Biological process analysis
CC: Cellular component analysis
CeRNA: Competing endogenous RNA
circRNAs: Circular RNAs
COPD: Chronic obstructive pulmonary disease
Dusp1: Dual Specificity Protein Phosphatase 1
FAM134B: Family with Sequence Similarity 134, Member B
FOXM1: Forkhead Box protein M1
FTO: Fat mass and obesity related protein
Glut1: Glucose Transporter 1
GO: Gene ontology analysis
Hes1: Hairy and Enhancer of Split 1
HIF-1α, HIF-2α: Hypoxia-inducible factor-1α,-2α
HPH: Hypoxia mediated pulmonary hypertension
Jun: Jun Proto-Oncogene AP-1 Transcription Factor Subunit
KEGG: Kyoto Encyclopedia of Genes and Genomes
KIAA1429: Vir like m6A methyltransferase associated
LncRNA: Long non-coding RNA
m^6^A: N6-methyladenosine
MeRIP: M^6^A RNA Immunoprecipitation
MeRIP-qRT-PCR: MeRIP-quantitative RT-PCR
METTL3, METTL14, METTL16: Methyltransferase-like-3, − 14 and − 16
MF: Molecular functions analysis
MREs: MiRNA response elements
Myc: Myc proto-oncogene bHLH transcription factor
ncRNAs: Noncoding RNAs
NMDAR1: N-methyl-D-aspartate receptor 1
PAECs: Pulmonary artery endothelial cells
PASMCs: Pulmonary artery smooth muscle cells
PDGF-BB: Platelet derived growth factor beta polypeptide b
PH: Pulmonary hypertension
PPαR: Peroxisome proliferator-activator
RBM15: RNA binding motif protein 15
RP11: RP11–138 J23.1
RVH: Right ventricular hypertrophy
RVSP: Right ventricular systolic pressure
WTAP: Wilms tumor 1-associated protein
YTHDF1, YTHDF2, YTHDF3, YTHDC1, YTHDC2: YT521-B homology domain-containing proteins family
ZC3H13: Zinc finger CCCH-type containing 13
